# Supplementary material for: Successful modulation of temporoparietal junction activity and stimulus-driven attention by fNIRS-based neurofeedback—A randomized controlled proof-of-concept study
Source: Imaging Neurosci (Camb). 2023 Sep 7;1:imag-1-00014. doi: 10.1162/imag_a_00014 (PMC12007532; doi:10.1162/imag_a_00014)
Supplement: Supplementary Material [file imag_a_00014-supp.pdf]

Supplementary Material

This document provides supplementary material pertaining to the manuscript “Successful Modulation of Temporoparietal Junction Activity and Stimulus-Driven Attention by fNIRS-based Neurofeedback – a Randomized, Controlled Proof-of-Concept Study”.

1 Neurofeedback instructions

See Supplementary file 2 instructions.pdf

2 CRED-nf checklist summary

See Supplementary file 3 CRED-nf checklist summary.pdf

Table S1. Questionnaire results and sample characteristics

	**Upregulation** *(M ± SD)*	**Downregulation** *(M ± SD)*	*p-value*
**PRE**			
N	27 (13 female)	18 (9 female)	
Age (years)	24.22 ± 3.03	24.22 ± 2.71	0.935
pre rTPJ thresholds	2.19 ± 1.45	2.76 ± 1.84	0.270
AQ total	15.30 ± 6.14	13.94 ± 4.49	0.399
EQ total	45.19 ± 9.76	45.17 ± 8.28	0.995
SQ total	29.96 ± 10.35	32.11 ± 13.75	0.577
ASRS: total	24.00 ± 9.57	19.94 ± 8.19	0.316
ASRS: inattention	12.22 ± 5.58	10.33 ± 4.86	0.236
ASRS: Hyp/Imp	11.78 ± 6.00	9.61 ± 4.46	0.172
IRI total[1]	56.78 ± 11.78	52.39 ± 8.83	0.161
IRI perspective-taking	20.07 ± 3.92	18.06 ± 2.94	0.055
IRI fantasy	16.78 ± 5.20	16.11 ± 5.12	0.673
IRI empathic concern	19.93 ± 4.51	18.22 ± 5.00	0.252
IRI personal distress	10.89 ± 2.94	9.61 ± 4.94	0.333
SRS: total	39.26 ± 19.36	37.94 ± 12.94	0.862
SRS: social awareness	5.00 ± 2.73	4.89 ± 2.03	0.921
SRS: social cognition	7.00 ± 3.99	6.06 ± 3.42	0.401
SRS: social communication	11.85 ± 9.07	10.83 ± 4.54	0.871
SRS: social motivation	7.89 ± 4.23	7.50 ± 3.54	1.000
SRS: autistic mannerism	7.11 ± 4.12	8.06 ± 3.59	0.334
Expectations	2.42 ± 0.56	2.67 ± 0.59	0.123
Motivation	3.70 ± 0.40	3.63 ± 0.50	0.695
**POST**			
Evaluation: belief efficacy	2.40 ± 0.87	2.39 ± 0.83	0.861
Evaluation: joy	2.13 ± 0.35	2.22 ± 0.40	0.431
Evaluation: experimenter	3.87 ± 0.26	3.81 ± 0.35	0.610
Neurofeedback control belief	7.59 ± 0.93	6.56 ± 1.54	0.020
Monetary reward	12.80 ± 2.33	7.86 ± 2.16€	< 0.001

[1] according to Cliffordson (2001) and Paulus (2012); AQ, Autism Spectrum Quotient; ASRS, Adult ADHD Self-Report Scale; EQ, Empathy Quotient; IRI, Interpersonal Reactivity Index; POMS, Profile of Mood States; SQ, Systemizing Quotient; SRS, Social Responsiveness Scale

Table S2. Descriptive statistics of pre-post experimental tasks

	Pre (*M ± SD*)	Post (*M ± SD*)
**Upregulation group**		
RTs attention task – invalid	497 ± 69 ms	481 ± 57 ms
RTs attention task – valid	452 ± 61 ms	433 ± 47 ms
Accuracies attention task – invalid	0.98 ± 0.02	0.98 ± 0.03
Accuracies attention task – valid trials	0.99 ± 0.02	0.99 ± 0.03
RTs vPT task – PT	3667 ± 327 ms	3520 ± 282 ms
RTs vPT task – NPT	3670 ± 286 ms	3490 ± 296 ms
Accuracies vPT task – PT	0.94 ± 0.07	0.98 ± 0.04
Accuracies vPT task – NPT	0.98 ± 0.04	0.99 ± 0.03
**Downregulation group**		
RTs attention task – invalid	508 ± 90 ms	521 ± 110 ms
RTs attention task – valid	468 ± 94 ms	486 ± 106 ms
Accuracies attention task – invalid	0.98 ± 0.05	0.98 ± 0.05
Accuracies attention task – valid trials	0.99 ± 0.03	0.99 ± 0.02
RTs vPT task – PT	3630 ± 302 ms	3500 ± 280 ms
RTs vPT task – NPT	3620 ± 272 ms	3480 ± 232 ms
Accuracies vPT task – PT	0.93 ± 0.09	0.98 ± 0.03
Accuracies vPT task – NPT	0.97 ± 0.04	0.99 ± 0.02

Note that we did not find a significant a three-way interaction of group × time × condition in the attention task or the vPT and only a group × time interaction in the attention task. Reaction times across conditions decreased in the upregulation group (pre = 474 ± 68ms, post = 457 ± 57ms, d = 0.51) and increased in the downregulation group across conditions (pre = 488 ± 93ms, post = 503 ± 108ms, d = -0.56; see main text). PT, perspective taking; NPT, non-perspective taking; vPT, visual perspective taking.

Table S3. POMS and general self-efficacy

	Pre	Post	*p-value*
	M ± SD or Md (IQR)		
**Upregulation group**			
General self-efficacy	30.59 ± 2.50	32.04 ± 3.16	0.068
POMS: depression/anxiety	0.21 (0.54)	0.14 (0.43)	0.104
POMS: vigor	3.43 (1.14)	3.29 (0.86)	0.592
POMS: fatigue	1.61 ± 0.99	1.81 ± 1.20	0.492
POMS: hostility	0.14 (0.21)	0.14 (0.21)	0.439
**Downregulation group**			
General self-efficacy	31.83 ± 3.57	32.00 ± 3.41	0.887
POMS: depression/anxiety	0.11 (0.63)	0.18 (0.29)	0.277
POMS: vigor	3.40 ± 0.90	2.94 ± 1.31	0.229
POMS: fatigue	1.86 (1.71)	1.57 (1.86)	0.228
POMS: hostility	0.07 (0.93)	0.21 (0.79)	0.627

Table S4. Motivation and self-control beliefs throughout the training

	Session 1	Session 2	Session 3	Session 4	*F*	*p*
	M ± SD or Md (IQR)					
**Upregulation group**						
Motivation	9 (2.25)	9 (2)	9 (2.25)	8 (3)	2.54	0.021*
Self-control belief	7.19±1.42	6.92±1.5	7.08±1.35	6.88±1.82	0.11	0.936
**Downregulation group**						
Motivation	9.5 (1.75)	9 (1.75)	9 (2)	9.5 (1.75)	0.681	0.48
Self-control belief	4.44±1.82	5.72±1.64	6.56±1.82	6.36±2.11	4.75	0.005**

3 Neurofeedback regulation success

3.1 Regulation behavior – durations above feedback thresholds

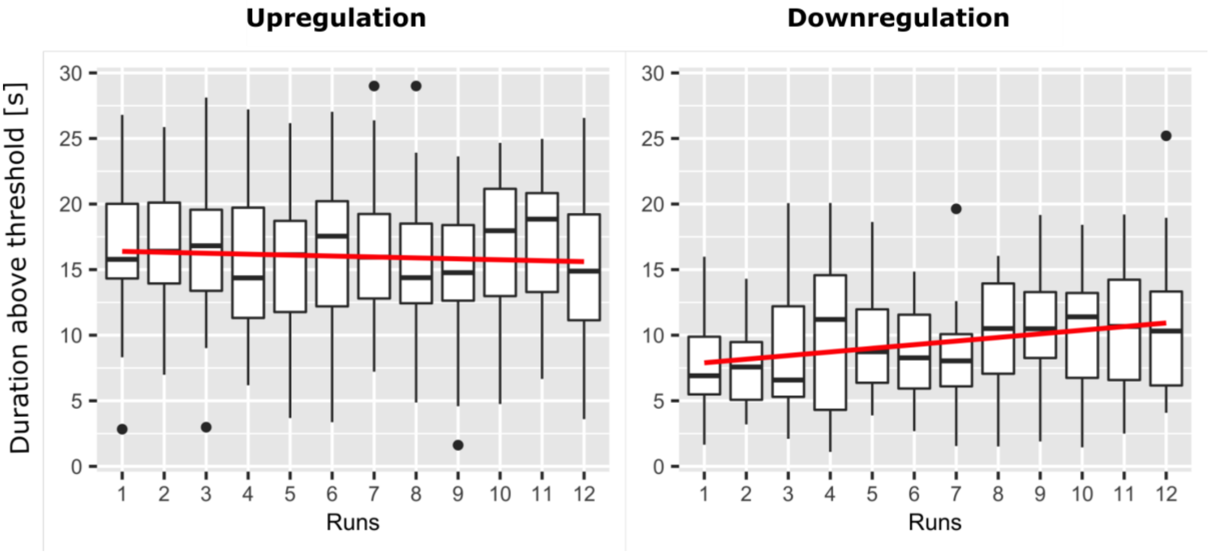

Figure S1. Development of feedback performance over time (maximum durations above the threshold). Box plots depicting the feedback performance as assessed by the maximum durations that participants could sustain the feedback signal above the individual threshold averaged over all participants for each run. The regression lines of the linear models are depicted in red.

Figure S1 shows the development of feedback performance over time (maximum durations above the threshold in a trial averaged over all participants for each run). Participants were able to keep the feedback signal above their individual thresholds and we found similar results to the main analysis of regulation success (analysis based on signal amplitudes). One-sample t-tests revealed a significant effect of regulation in the upregulation group (M = 16±3.25s, t(26) = 25.59, p < 0.001, d = 5.02) and downregulation group (M = 9.41±2.9s, t(17) = 13.76, p < 0.001, d = 3.34). Paired-sample t-tests only revealed a difference between the last and the first session in the downregulation group ($Mdiff$ = 3.2±4.78, t(17) = 2.83, p = 0.01, d = 0.56). This was not the case in the upregulation group ($Mdiff$ = -0.86±5.89s, p = 0.46, d = 0.15). Mixed models revealed a significant effect of groups (F(1,43) = 48.25, p < 0.001, η_p^2 = 0.53) as well as a marginal group × time effect (F(11,473) = 1.72, p = 0.07, η_p^2 = 0.04) and time effect (F(11,473) = 1.53, p = 0.12, η_p^2 = 0.03). There was no significant within-group time effect in either group. In the upregulation group, 40.7% of the participants (11 of 27) showed increasing durations above the threshold over trials, as indicated by a positive slope of the regression, and 16 of 27 participants (59.26%) showed longer durations

above the threshold in the last session compared to the first session. In the downregulation group, 61.1% of the participants (11 of 18) showed increasing durations above the threshold over trials, as indicated by a positive slope of the regression and twelve of 18 participants (66.7%) showed longer durations above the threshold in the last session compared to the first session.

3.2 Dynamic of the feedback signal – switching between above and below the threshold

We also explored the dynamics of the feedback signal by analyzing the number of switches between below and above the feedback threshold, but observed no significant effects (see Figure S2). Mixed models revealed no significant interaction effect or main effect of time. On average, participants switched 1.92 times per trial (upregulation group = 2 ± 0.10, downregulation group = 1.80 ± 0.18 times). Paired-sample t-tests comparing the last and the first session also revealed no effect for either group. In the upregulation group, 48.15% of the participants (13 of 27) showed decreasing number of switches between below and above the threshold over trials, as indicated by a negative slope of the regression, and 51.85% (14 of 27 participants) showed less switching in the last session compared to the first session. In the downregulation group, 55.56% of the participants (10 of 18) showed decreasing switches over trials, as indicated by a negative slope of the regression, and 38.89% (7 of 18 participants) showed less switching in the last session compared to the first session.

In total, the number of switches between above and below a feedback threshold did not reveal a learning effect. On average, participants did not cross the feedback threshold very often (only 1-2 times per trial), which made this measure unsuitable for detecting a learning effect. However, the sensitivity of time-based measures depends on the appropriate estimation and selection of feedback thresholds used for the calculation (see limitations).

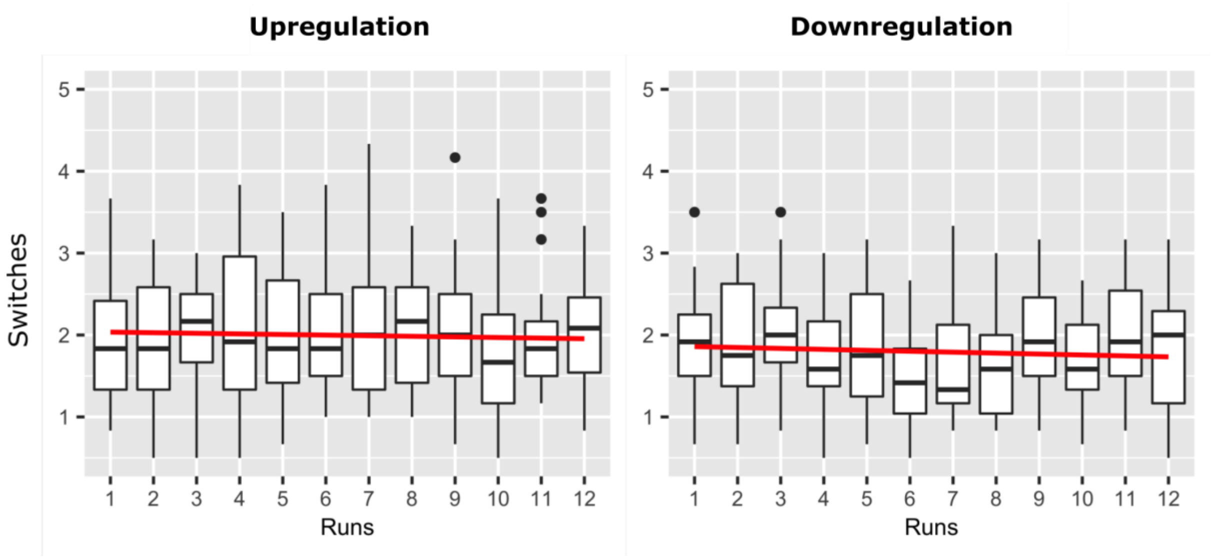

Figure S2. Dynamic of the feedback signal. Box plots depicting the number of switches between above and below the threshold averaged over all participants for each run. The regression lines of the linear models are depicted in red.

Table S5. Individual neurofeedback success in the upregulation group

Subject	Succesful runs		Last vs. first		Linear slope	
U1	7	Yes	-2.28	No	-0.09	No
U2	12	Yes	3.00	Yes	0.21	Yes
U3	10	Yes	-3.51	No	-0.32	No
U4	8	Yes	0.70	Yes	0.00	Yes
U5	10	Yes	3.91	Yes	0.13	Yes
U6	9	Yes	5.16	Yes	0.62	Yes
U7	8	Yes	0.79	Yes	-0.13	No
U8	11	Yes	-3.84	No	-0.36	No
U9	7	Yes	-7.18	No	-0.59	No
U10	12	Yes	0.76	Yes	0.30	Yes
U11	12	Yes	2.56	Yes	0.17	Yes
U12	12	Yes	4.46	Yes	0.43	Yes
U13	12	Yes	2.39	Yes	0.16	Yes
U14	10	Yes	0.63	Yes	0.00	No
U15	10	Yes	-4.92	No	-0.41	No
U16	5	No	7.75	Yes	0.81	Yes
U17	8	Yes	-1.75	No	-0.13	No
U18	11	Yes	-1.70	No	0.03	Yes
U19	11	Yes	-6.86	No	-0.34	No
U20	12	Yes	3.93	Yes	0.16	Yes
U21	12	Yes	2.10	Yes	0.16	Yes
U22	7	Yes	1.54	Yes	0.00	Yes
U23	12	Yes	-3.00	No	-0.26	No
U24	7	Yes	-0.17	No	-0.25	No
U25	7	Yes	1.75	Yes	0.45	Yes
U26	6	Yes	-3.43	No	-0.44	No
U27	12	Yes	-2.14	No	-0.10	No
	M = 9.63	26/27	M = 0.02 ± 3.7	15/27	M = 0.01 ± 0.34	14/27

Table S6. Individual neurofeedback success in the downregulation group

Subject	Succesful runs		Last vs. first		Linear slope	
D1	2	No	-2.18	Yes	-0.04	Yes
D2	6	Yes	-2.93	Yes	-0.04	Yes
D3	6	Yes	-8.68	Yes	-0.20	Yes
D4	2	No	-0.14	Yes	-0.03	Yes
D5	0	No	-2.03	Yes	-0.06	Yes
D6	0	No	-0.24	Yes	-0.01	Yes
D7	5	No	-1.51	Yes	0.02	No
D8	0	No	-1.05	Yes	-0.03	Yes
D9	9	Yes	-8.99	Yes	-0.09	Yes
D10	4	No	-1.28	Yes	-0.01	Yes
D11	3	No	-8.40	Yes	-0.12	Yes
D12	3	No	-2.35	Yes	-0.03	Yes
D13	0	No	-4.32	Yes	-0.06	Yes
D14	0	No	-0.91	Yes	-0.03	Yes
D15	3	No	0.51	No	-0.03	Yes
D16	6	Yes	0.34	No	0.01	No
D17	4	No	-3.91	Yes	-0.04	Yes
D18	1	No	5.55	No	0.08	No
	M = 3	4/18	M = -2.36 ± 3.6	15/18	M = -0.22 ± 0.35	15/18

3.3 Robustness checks

Figure S3 shows the regulation performance based on the robustness checks, Table S7 statistical results and figure S4 the grand averages based on the different analyses approaches.

Robustness check 1: Bandpass filter (0.01-0.09Hz)

Robustness check 1 confirmed the results of the online analysis. One-sample t-tests again revealed a significant effect of regulation in the upregulation group (*M* = 5.02±4.74, t(26) = 5.51, $p < 0.001$, $d = 1.08$) and downregulation group (*M* = 2.39±4.28, $t(17) = 2.37$, $p = 0.03$, d = 0.58). Paired-sample t-tests only revealed a difference between the last and first session in the downregulation group (*Mdiff* = -6.22±7.76, $t(17) = 2.79$, $p = 0.01$, $d = 0.82$). This was not the case in the upregulation group (*Mdiff* = -1.03±8.59, $p = 0.54$, $d = 0.12$). The non-parametric ANOVA revealed a marginal group effect ($F_{ATS}(1, \infty) = 3.33$, $p = 0.07$), no significant group × time interaction, and a significant time effect ($F_{ATS}(8.17, \infty) = 2.23$, p =

0.02). Separate analysis for both groups showed no effect in the upregulation group and only a non-significant time trend in the downregulation group (F(11,204) = 1.59, p = 0.1, η_p^2 = 0.08).

Robustness check 2: Common average reference

On the group level, none of the effects survived the more conservative robustness check 2. One-sample t-tests revealed no significant effect of regulation in the upregulation group (M = -0.15±2.76, p = 0.78, d = -0.06), and no significant effect in the downregulation group (M = 0.11±4.68, p = 0.93, d = 0.02). Paired-sample t-tests revealed no difference between the last and the first session in both groups. The learning effect for the downregulation group disappeared ($Mdiff$ = -1.25±5.28, p = 0.329, d = 0.24). The non-parametric ANOVA revealed no group effect and no significant group × time interaction. The trend effect of time disappeared (both groups: F_{ATS} (8.4, ∞) = 0.87, p = 0.52; downregulation group: F_{ATS} (6.68, ∞) = 0.91, p = 0.5).

Table S7. Neurofeedback regulation success – offline robustness checks

	**Offline robustness check 1 (BP 0.01-0.09Hz)**			**Offline robustness check 2 (CAR)**		
**Upregulation group**						
	M±SD	*N*	*p-value*	*M±SD*	*N*	*p-value*
NF performance - compared to baseline	5.02±4.74	25/27	0.001*	-0.15±2.76	15/27	0.612
NF improvement (slope)	-0.11±0.83	11/27	0.25	-0.02±0.45	12/27	0.43.
NF improvement (last vs first)	2.39±4.28	13/27	0.54	0.20±5.39	13/27	0.845
**Downregulation group**						
	M±SD	*N*	*p-value*	*M±SD*	*N*	*p-value*
NF performance - compared to baseline	2.39±4.28	7/18	0.99	0.11±4.68	10/18	0.537
NF improvement (slope)	-0.40±0.76	14/18	0.1	-0.12±0.47	14/18	0.496
NF improvement (last vs first)	-6.22±7.76	14/18	0.003*	-1.25±5.28	11/18	0.329

Neurofeedback regulation success according to different success measures and offline robustness checks for both groups. The p-values reflect the results of the group analysis based on the description in 2.5 "Data processing and analysis"; NF, neurofeedback

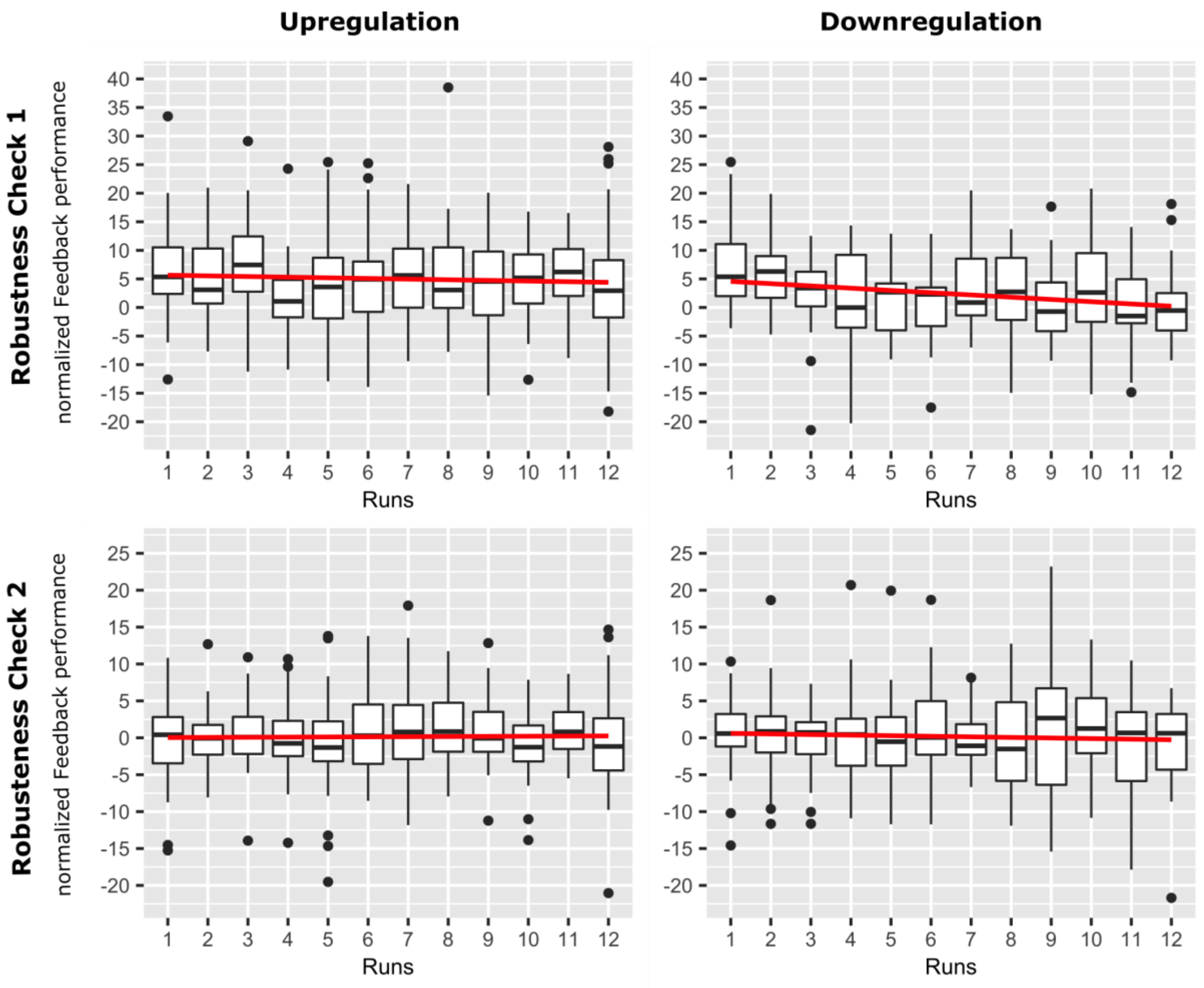

Figure S3. Neurofeedback regulation performance based on an additional offline analysis using more stringent artifact correction methods (robustness checks). The first row shows box plots of the average feedback performance as assessed by the standardized median change of rTPJ activation averaged over participants for all runs based on robustness check 1 (stronger bandpass filter 0.01-0.09Hz, see methods section). The second row shows box plots of the average feedback performance as assessed by the standardized median change of rTPJ activation averaged over participants for all runs based on robustness check 2 (common average correction, see methods section). The regression lines of the linear models are depicted in red.

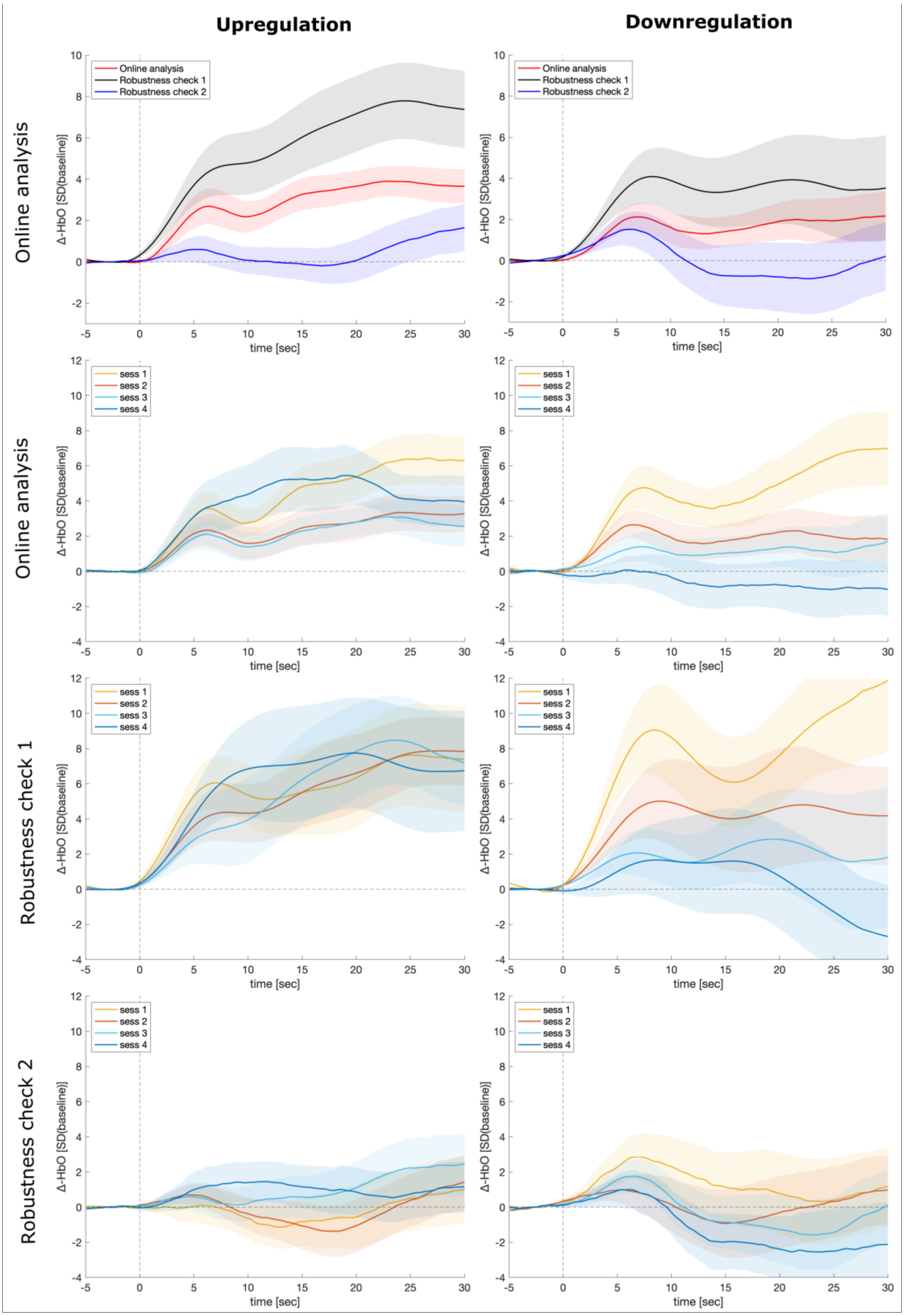

Figure S4. Grand averages of the feedback channel for the sessions of both groups. The four rows show the grand averages ± standard error (shaded area) for the different offline

analysis approaches based on the raw fNIRS signal: (1) offline analysis mimicking the online analysis, (2) analysis with a more stringent bandpass filter, (3) common average reference (CAR) approach. All analysis approaches included the correlation-based signal improvement (CBSI; Cui et al., 2010) and a 5s moving average filter. Row 1: grand averages over all sessions of the three analysis approaches; Row 2-4 session-wise grand averages for the three analysis approaches.

4 Results for the reorienting of attention task including valid only blocks

The pre-post comparisons revealed that after the neurofeedback training, reaction times decreased in the upregulation group (pre = 468 ± 71ms, post = 450 ± 61ms, $d = 0.58$) and increased in the downregulation-group across conditions (pre = 480 ± 94ms, post = 493 ± 107ms, $d = -0.49$), as indicated by a significant group × time interaction ($F(1,123) = 15.14$, $p < 0.001$) and a significant main effect of time in the upregulation group ($F_{ATS}(1, \infty) = 10.01$, $p = 0.001$) as well as a marginally significant effect of time in the downregulation group ($F_{ATS}(1, \infty) = 3.25$, $p = 0.071$). No three-way interaction of group × time × condition was observed.

5 Mental strategies underlying neurofeedback regulation

Table S8 shows the results for the reported mental strategies and their success ratings for both groups relative to the total number of strategies reported by each group. In total, mental strategies for 1299 trials were reported (771 by the upregulation group and 528 by the downregulation group). Socio-cognitive strategies and positive mental imagery were reported the most in both groups. Both groups seemed to be equally reliant on socio-cognitive strategies (upregulation group: 15.82% vs. downregulation group: 15.34), with the upregulation group reporting socio-cognitive strategies as more effective than the downregulation group (mean rating: 4.03 vs. 2.28). The upregulation group used more positive mental imagery, arithmetic strategies, and music-related strategies than the downregulation group. The downregulation group used slightly more relaxation strategies and strategies related to memory and focused attention.

Most strategies seemed to work better in the upregulation group (mean success rating of 3.35) than in the downregulation group (mean success rating of 2.74). Socio-cognitive, arithmetic, and working memory strategies seemed to perform better in the upregulation group. Moreover, the upregulation group reported positive and negative mental imagery, language, music, and visual imagery as slightly more effective. The downregulation group reported thinking, memory, and relaxation strategies as slightly more effective. In summary, both groups did not differ much regarding the use of mental strategies, but some strategies seemed to work better depending on the regulation condition.

Table S8. Reported mental strategies and their success ratings for both groups

	Upregulation group		Downregulation group	
	Strategies reported [%]	Mean rating	Strategies reported [%]	Mean rating
Socio-cognitive strategies	15.40	4.11	15.34	2.28
Positive mental imagery	24.05	3.42	16.86	2.99
Language	6.45	3.43	6.25	3.05
Counting	6.60	2.98	5.11	2.59
Arithmetic	9.24	3.31	5.68	2.10
Working memory	5.72	3.32	5.49	3.03
Planning	2.35	2.88	1.70	3.39
Desire to regulate and increase feedback signal	4.69	3.25	5.49	3.14
Out-of-body imagination	2.79	2.71	4.92	2.65
Music	6.60	3.01	3.98	2.71
Relaxation	2.79	3.08	8.33	3.45
Negative mental imagery	0.59	2.75	1.14	2.42
Motor imagery	2.35	2.56	1.70	2.78
Connection to avatar	1.47	3.05	N/A	N/A
Thinking	0.44	1.67	1.33	2.71
Memory	1.61	3.09	4.73	3.40
Focused attention	0.59	2.75	5.30	2.77
Visual imagery	5.13	3.44	4.73	2.70
Sensory imagery	N/A	N/A	0.38	2.50
Mindfulness	N/A	N/A	0.95	4.20
Uncategorized	1.17	2.50	0.57	3.67
**Total**		3.35		2.84

6 Correlations of behavioral outcomes with regulation performance and psychosocial factors

Table S9. Significant correlations between behavioral outcomes and regulation success measures with and without multiple comparison correction

Group	Behavioral outcome	Regulation success measure	Rho	p uncorrected	p adjusted
both	**Δ** RT attention	successful runs	-0.38	0.012	0.321
both	**Δ** RT attention - valid	succesful runs	-0.47	0.002	0.045*
both	**Δ** accuracy perspective taking	slopes	-0.38	0.012	0.327
both	**Δ** accuracy perspective taking - NPT	slopes	-0.49	0.001	0.021*
up	**Δ** RT attention	slopes	0.41	0.041	1.000
up	**Δ** RT attention - invalid	slopes	0.39	0.047	1.000
up	**Δ** accuracy perspective taking	successful runs	-0.39	0.045	1.000

up	**Δ** accuracy perspective taking - PT	successful runs	-0.42	0.030	0.799
down	**Δ** accuracy perspective taking - NPT	slopes	-0.71	0.001	0.039*

* significant after Bonferroni–Holm correction; NPT, non-perspective taking trials; PT, perspective taking trials.

Table S10. Significant correlations between behavioral outcomes and psychosocial factors with and without multiple comparison correction

Group	Behavioral outcome	Psychosocial factor	rho	p uncorrected	p adjusted
both	**Δ** RT attention	NF control belief	-0.3	0.048	1.000
both	**Δ** RT attention	Monetary reward	-0.36	0.020	0.650
both	**Δ** accuracy perspective taking	Expectations	0.33	0.027	0.894
up	**Δ** RT attention	NF control belief	-0.5	0.041	1.000
down	**Δ** RT attention	Evaluation: experimenter	-0.46	0.019	0.637

* significant after Bonferroni–Holm correction; NF, neurofeedback

Neurofeedback instructions (translated from German)

During this task you will receive Feedback of the activation of a certain brain region with the goal for you to learn how to control it. The feedback will be delivered in the form of a smiling avatar. The better you are at regulating, the more the avatar will smile.

One neurofeedback run consists of 6 block. There will be 2 to 4 runs during each session.

A block consists of a regulation and a non-regulation condition.

During the non-regulation condition, you will see the avatar with a neutral facial expression. You will not receive any feedback, so the avatar will not smile. Next to the avatar you will see two + signs, which indicate the condition. Please, try not to regulate your brain activity during this condition. Just look at the avatar, relax and don’t think about anything specific.

After that the non-regulation condition will start, which you will notice through two 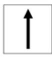 appearing next to the avatar. Try now to regulate your brain activity by using mental strategies and make the avatar smile.

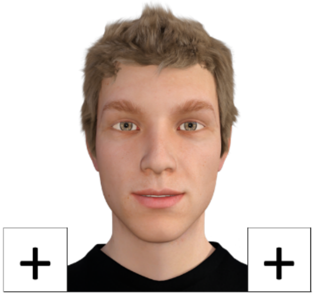

Nicht regulieren
und entspannen (20-25 sec)

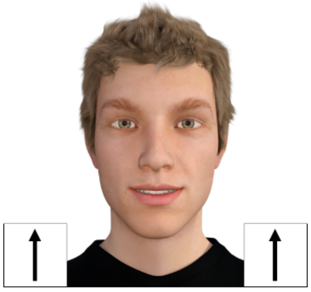

Regulierung (30 sec)

Depending on how good you are at regulating your brain activity you can earn additional money.

As soon as your brain activity passes a certain threshold a green frame will appear around the avatar. For each second that you are able to remain above this threshold you will earn 0.01€. At the end of each block you will see how much you have earned.

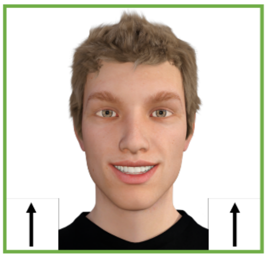

Strategies

As already said, you should use mental strategies to regulate your brain activity.

It is important that you find your own individual strategy to succeed. These can be different for everyone. Here are a few examples that you could try to start with:

- Try to put yourself into the avatar. What is he currently thinking or feeling?
- Think about what he is doing in his life. For example, what is his job? What is he studying? What kind of hobbies does he have? What did he do yesterday? How was his day?
- Imagine you can make the avatar smile with your own mental states
- Think about you being the avatar
- Think about leaving your body
- Think about positive life events
- Imagine a simple arithmetic problem
- Count backwards in steps of 7.
- Imagine words and read them backwards

If these do not work for you, you can also come up with your own strategies and try them out. Anything that helps to make the avatar smile is good.

Note that the signal is delayed about 4-6 seconds. So use only one strategy during one block and don't switch in between.

Again, please only use mental strategies. Stay calm and relaxed and breath regularly.

Good Luck!

Neurofeedback instruction (in German)

In dieser Aufgabe erhältst du Rückmeldung über die Gehirnaktivierung eines bestimmten Areals und sollst lernen diese zu beeinflussen. Die Rückmeldung wird dir in From eines lächelnden Avatars gegeben. Je besser du regulierst, desto mehr lächelt der Avatar.

Ein Neurofeedback-Durchgang besteht aus 6 Blöcken. Insgesamt besteht das Neurofeedback aus 2-4 Durchgängen.

Ein Block besteht jeweils aus einer Nichtregulierungs- und Regulierungsbedingung.

Während der Nichtregulierungsbedingung siehst du den Avatar mit neutralem Gesichtsausdruck. Du erhältst in dieser Bedingung kein Feedback, der Avatar wird also nicht lächeln. Neben dem Avatar siehst du zwei + Zeichen, die dir die Bedingung anzeigen. Bitte versuche in dieser Bedingung nicht deine Gehirnaktivität zu regulieren. Schaue einfach den Avatar an, entspanne dich und denke an nichts bestimmtes.

Danach beginnt die Regulierungsbedingung. Das erkennst du daran, dass zwei 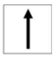 neben dem Avatar erscheinen. Versuche jetzt mittels mentaler Strategien, deine Hirnaktivierung zu regulieren und den Avatar zum Lächeln zu bringen.

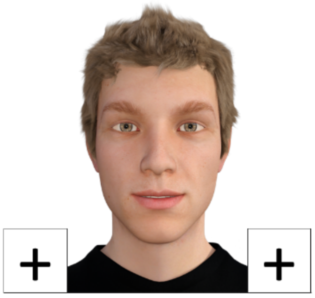

Nicht regulieren
und entspannen (20-25 sec)

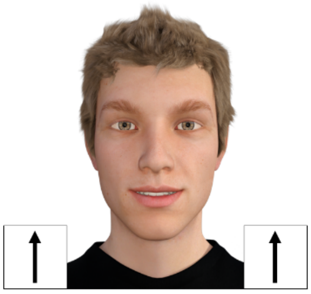

Regulierung (30 sec)

Je nachdem wie gut du regulieren kannst, kannst du in dieser Aufgabe Geld dazugewinnen.

Sobald deine Hirnaktivierung eine bestimmte Schwelle überschritten hat erscheint ein grüner Rahmen um den Avatar. Für jede Sekunde, die es dir gelingt, über dieser Schwelle zu bleiben, erhälst du 0,01 €. Am Ende eines jden Blocks wir dir dann angezeigt, wieviel du gewonnen hast.

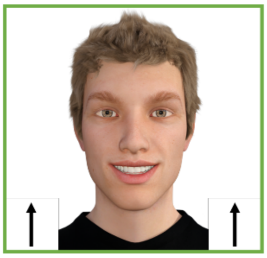

Strategien

Wie schon gesagt, sollst du mentale Strategien nutzen, um deine Hirnaktivierung zu regulieren.

Wichtig ist, dass du deine eigene für dich erfolgreiche Strategie findest. Das kann bei jedem eine andere sein. Hier einige Beispiele, die du am Anfang ausprobieren kannst:

- Sich in den Avatar hineinversetzen. Zum Beispiel überlegen, was dieser gerade denkt oder wie er sich fühlt.
- Überlegen, was der Avatar macht. Z.B was macht er beruflich? Was studiert er? Was hat er für Hobbies? Was hat er gestern gemacht, wie war sein Tag?
- Die Vorstellung, den Avatar mit Hilfe der eigenen mentalen Zustände zum lächeln zu bringen.
- Vorstellung, dass man selber der Avatar ist.
- Vorstellung, dass man seinen Körper verlässt.
- Sich positive eigene Lebensereignisse vorstellen.
- Einfache Rechenaufgabe vorstellen.
- Zählen, z.B. in 7er Schritten Rückwarts zählen.
- Wörter vorstellen, die man rückwärts liest.

Falls diese nicht funktionieren, kannst du dir aber auch eigene Strategien überlegen und diese ausprobieren. Wichtig ist, dass der Avatar lächelt.

Das Signal ist um 4-6 Sekunden verzögert. Probiere also pro Block (30sek) nur eine Strategie aus und wechsele nicht mittendrin.

Nochmal zur Erinnerung: Nutze nur mentale Strategien. Bleibe ruhig und entspannt sitzen und atme regelmäßig.

Viel Erfolg!

CRED-nf checklist summary

11 July, 2023

Manuscript title: Successful Modulation of Temporoparietal Junction Activity and Stimulus-Driven Attention by fNIRS-based Neurofeedback – a Randomized Controlled Proof-of-Concept Study
Corresponding Author: Simon H. Kohl
Corresponding author email: simon.h.kohl@gmail.com

Item No.	Checklist item	Manuscript Details
**Pre-experiment**		
1a	Pre-register experimental protocol and planned analyses	*This experiment was not preregistered*
1b	Justify sample size	This was a proof-of-concept study. Hence, no a priori power analysis was conducted. However, according to a sensitivity analysis, a mixed analysis of variance (ANOVA) including 45 participants was sufficiently powered (80%) to detect a group x time interaction effect of at least f = 0.43 (assuming no violation of sphericity and a correlation among repeated measure of 0.8) or 0.77 for an independent t-test.
**Control groups**		
2a	Employ control group(s) or control condition(s)	In a bidirectional regulation control group design, 50 healthy participants were either reinforced to up- or downregulate rTPJ activation over four days of training.
2b	When leveraging experimental designs where a double-blind is possible, use a double-blind	*The experiment did not include a double-blind*
2c	Blind those who rate the outcomes	*Those who rated the outcome were not blind to group assignment*
	Blind those who analyse the data	*Those who analysed the data were not blind to group assignment*
2d	Examine to what extent participants and experimenters remain blinded	Furthermore, they were asked to guess the group condition they had been randomly assigned to.
2e	In clinical efficacy studies, employ a standard-of-care intervention group as a benchmark for improvement	*NA: This is not a clinical efficacy study*

3a	Collect data on psychosocial factors	At the end of the session, participants filled in the general self-efficacy scale again as well as a debriefing questionnaire to further assess feasibility and unspecific mechanisms. This questionnaire included items assessing participants' evaluation of the neurofeedback training, for example "I believe the training helped to improve my attention", "I enjoyed the training", "The experimenter was trustworthy", etc. Furthermore, they were asked to guess the group condition they had been randomly assigned to. [...] After each session, we also assessed participants' motivation to continue participating in the training and their beliefs about being able to control their brain activity. [...] Regarding non-specific mechanisms, we were unable to find between-group differences in expectation towards the neurofeedback training and with respect to the evaluation of the training.
3b	Report whether participants were provided with a strategy	we provided some example strategies that could be helpful to regulate rTPJ activity (e.g., strategies related to ToM, empathy, thinking, imagination of positive events, counting, etc.; see Supplementary Material 1). However, participants were encouraged to find their own individual successful strategy by trial and error. After each neurofeedback run, we asked participants to verbally report which strategies they used and how successful they rated this strategy (Likert scale ranging from 1 to 5).
3c	Report the strategies participants used	The downregulation group used significantly more different strategies ($M = 8.66 \pm 2.47$) during the neurofeedback training compared to the upregulation group ($M = 6.26 \pm 3.24$; $t(42.11) = 2.82$, $p = 0.007$, $d = 0.87$). Figure 7 shows the distribution of strategies as reported by the participants of both groups. Fisher's exact Chi-square test revealed no significant association between the group and reported strategies ($p = 0.982$), indicating that similar strategies were used for both upregulating and downregulating TPJ activity. Table S8 shows the percentages of strategies relative to the total number of strategies reported per group and their mean success rating. In total, most strategies were reported to be more successful in the upregulation group (mean success rating: 3.35) than in the downregulation group (mean success rating: 2.74), and socio-cognitive strategies and positive mental imagery were reported most frequently in both groups (see Supplementary Material 5).
3d	Report methods used for online-data processing and artifact correction	See section: Real-time fNIRS data processing (online analysis)
3e	Report condition and group effects for artifacts	*Condition and group effects for artifacts were not measured, or not reported in the manuscript*
**Feedback specifications**		
4a	Report how the online-feature extraction was defined	See section: Real-time fNIRS data processing (online analysis)

4b	Report and justify the reinforcement schedule	Whenever participants exceeded this reward threshold, a green frame appeared around the feedback display, indicating that their regulation was earning an incentive. The total amount earned on each trial was presented on the screen at the end of the trial. This threshold was adapted according to individual regulation performance (see 2.4. for a detailed description).
4c	Report the feedback modality and content	During the no-regulation condition, participants were instructed to passively look at the avatar, which maintained a neutral facial expression. During the regulation condition, real-time feedback of rTPJ activity was presented visually on a screen using a smiling avatar (social reward). Participants were instructed to regulate and make the avatar smile, which was modulated in real time by their rTPJ activation.
4d	Collect and report all brain activity variable(s) and/or contrasts used for feedback, as displayed to experimental participants	See results section: 3.3 Neurofeedback regulation success
4e	Report the hardware and software used	See sections: 2.3 fNIRS acquisition and 2.4 Real-time fNIRS data processing (online analysis) and subsection Statistical methods and software of section 2.5 Data processing and analysis
**Outcome measures - brain**		
5a	Report neurofeedback regulation success based on the feedback signal	see results section: 3.3 Neurofeedback regulation success
5b	Plot within-session and between-session regulation blocks of feedback variable(s), as well as pre-to-post resting baselines or contrasts	See Figure 5, S1, S2 and S4
5c	Statistically compare the experimental condition/group to the control condition(s)/group(s) (not only each group to baseline measures)	No specific group effect or significant group × time interaction was found.
**Outcome measures - behaviour**		
6a	Include measures of clinical or behavioural significance, defined a priori, and describe whether they were reached	*The manuscript does not include measures of clinical or behavioural significance*

6b	Run correlational analyses between regulation success and behavioural outcomes	We found a significant negative correlation between changes in RTs in the valid trials of the reorienting of attention task and neurofeedback performance, as assessed by the number of successful runs (rho = -0.47, p = 0.045, Bonferroni corrected), indicating higher improvements of RTs in participants with more successful runs in both groups. Subgroup analysis revealed no significant effect after Bonferroni correction. For the perspective-taking task, we found a significant correlation between neurofeedback improvement (slopes) and improvements in the accuracies of NPT trials across groups (rho = -0.49, p = 0.02), indicating greater performance improvements in participants who were more successful in learning downregulation over the course of the training. This significant correlation was only observed in the downregulation group (rho = -0.71, p = 0.039, Bonferroni corrected). None of the psychosocial factors correlated significantly with behavioral outcomes after Bonferroni correction. For more details including significant correlations on the uncorrected level, see Supplementary Material 6.
**Data storage**		
7a	Upload all materials, analysis scripts, code, and raw data used for analyses, as well as final values, to an open access data repository, when feasible	*No additional documents related to the materials, analysis scripts, code, raw data, or final values are available for this manuscript*
